# Towards automated dose‐guided patient positioning at clinical timescale in head‐and‐neck cancer proton radiotherapy

**DOI:** 10.1002/mp.70591

**Published:** 2026-07-28

**Authors:** Femke Oosterhof, Gabriel Guterres Marmitt, Alexander M. Kamerbeek, Jeffrey Free, Gerolf Meulman, Zohreh Shahpouri, Pietro Pisciotta, Johannes A. Langendijk, Stefan Both

**Affiliations:** ^1^ Department of Radiation Oncology University Medical Center Groningen, University of Groningen Groningen The Netherlands; ^2^ Department of Radiotherapy Isala Zwolle The Netherlands; ^3^ Institute for Life Science & Technology Hanze University of Applied Sciences Groningen The Netherlands

**Keywords:** dose‐guided patient positioning, head and neck cancer, proton radiotherapy

## Abstract

**Background:**

In intensity modulated proton therapy (IMPT) accurate patient positioning prior to treatment delivery is important to ensure that the prescribed dose is delivered as intended. Typically, in‐room 3D imaging is utilized to position the patient based on anatomical features, which can be challenging due to interfractional anatomical changes.

**Purpose:**

This study aims to introduce a fast automated in‐room dose‐guided patient positioning (DGPP) algorithm and to evaluate its performance for obtaining accurate target coverage in head‐and‐neck cancer (HNC) patients.

**Methods:**

The DGPP algorithm employs Moqui, an open‐source Monte Carlo (MC) dose engine, for fast proton dose computations. A beam model was configured specific to our institute's treatment machine using measured beam data and it was compared to the clinical treatment planning system (TPS) by means of a 2%/2 mm gamma analysis of 45 HNC treatment plan dose computations. The same cohort of 45 HNC patients, each with five to seven weekly repeat CTs (rCTs) acquired for longitudinal treatment quality assurance, was used to evaluate DGPP performance. For each rCT, the patient position relative to the plan isocenter was optimized using gradient descent to maximize the V95% of the primary CTV, 70 Gy(RBE), and elective CTV, 54.25 Gy(RBE), with a 1 mm margin applied to the CTVs during optimization. Six initial positions per rCT were considered, randomly generated within ±7 mm in each translational directions relative to the anatomy‐based alignment. The resulting V95% values were compared with those obtained using anatomy‐based patient positioning (ABPP) in the clinical TPS by evaluating the difference in V95% (ΔV95%).

**Results:**

The 3D 2%/2 mm gamma pass rate of Moqui dose computations, with as reference the doses computed in the clinical TPS, was at least 99.1%. By employing DGPP to optimize the rCT positions we found ΔV95%=(0.1±0.3)% and ΔV95%=(−0.1±0.4)% for the CTV 70 Gy(RBE) and CTV 54.24 Gy(RBE), respectively. Considering only the cases that did not reach the clinical goal V95%≥98% with ABPP, the V95% CTV 70 Gy(RBE) improved with (1.0 ± 0.8)%. The average optimization time was (149 ± 51) seconds.

**Conclusions:**

We demonstrated that with a gradient descent algorithm for DGPP using a GPU MC proton dose engine, accurate automated HNC patient alignment to the CTV is possible at near‐clinically acceptable timescale for pre‐treatment patient positioning. Further work is warranted to facilitate clinical adoption.

## 1 INTRODUCTION

With intensity modulated proton radiotherapy (IMPT) it is possible to deliver a highly conformal dose distribution to the target volume and to spare surrounding healthy tissues compared to photon radiotherapy, attributable to the physical characteristics of protons.^1^, ^2^, ^3^ The proton dose distributions are, however, sensitive to density variations and, consequently, to patient positioning inaccuracies and anatomical changes during the treatment course.^4^ For head‐and‐neck cancer (HNC) patients interfractional changes due to factors such as weight loss, tumor shrinkage, or variation in patient setup can significantly affect the delivered dose distribution,^5^, ^6^, ^7^, ^8^ which emphasizes the need for optimized patient positioning prior to each treatment fraction.

Patient positioning is typically performed using in‐room imaging. The use of in‐room 3D imaging devices, such as a gantry‐mounted cone‐beam computed tomography (CBCT) system, is becoming standard in proton therapy.^9^ Patients are positioned by registering the daily image to the treatment planning CT (pCT) based on anatomical structures, most commonly bones or markers. This rigid registration involves three to six degrees of freedom (DOFs), with the number of DOFs depending on whether the treatment couch can perform rotations (yaw, pitch, roll) in addition to translations (craniocaudal, lateral, anteroposterior). In the presence of non‐rigid anatomical changes, an exact rigid registration cannot be performed and it can be unclear which position results in the optimal dose distribution.

Using in‐room 3D imaging, the treatment dose distribution can be computed on the daily patient anatomy,^10^, ^11^, ^12^ enabling patient positioning guided by the dose to be delivered to the patient.^13^ The potential of dose‐guided patient positioning (DGPP) in proton therapy was first examined for lung cancer patients using a fast dose approximation method to determine the dosimetric parameters of interest on a grid of 729 treatment isocenter positions.^13^ Improved target coverage could be obtained. A multicriteria optimization algorithm to optimize patient position based on dose following a set of predefined clinical goals,^14^ was successfully applied in a retrospective study for prostate and HN cancer patients treated with proton therapy.^15^ Linear dose interpolation was used to scan a continuous space of potential isocenter shifts between 13 isocenter positions for which the dose was computed using a pencil‐beam algorithm. Enhanced sparing of serial organs at risk (OARs) for HNC patients and improved target coverage for prostate cancer patients compared to bony‐anatomy‐based alignment could be obtained.

Well commissioned Monte Carlo (MC) algorithms are more accurate for proton dose computation than analytic methods, especially in complex geometries or for beams with a range shifter and a large airgap.^16^, ^17^, ^18^, ^19^ To be used online for DGPP, the dose computation algorithm must be sufficiently fast to avoid prolonging of patient treatment session time. In the recent years, advances in graphic processing unit (GPU) accelerated MC algorithms improved the time efficiency of the proton dose computation, while with the advent of AI new opportunities still exist.^20^

Efforts are also being made towards online adaptive proton therapy, in which the clinical treatment plan is reoptimized on the daily image before treatment delivery to enhance tumor control and/or normal tissue sparing.^21^, ^22^, ^23^ Online adaptive radiotherapy is, however, resource intensive since it requires reviewing of online‐generated contours and verification of adapted treatment plans by trained specialists.^24^ Dose‐guided patient position, in combination with a fast offline plan adaption workflow, could be an interesting alternative when no clinical benefit is expected from online versus offline plan adaptation or when available resources are limited. It could also supplement a workflow in which online plan adaptation is only performed when needed, as assessed on the daily image.

In this paper, we present a fast algorithm for automated DGPP in IMPT.^25^ It uses a fast MC proton dose engine, Moqui,^26^ which was validated for our institute's treatment machine, in combination with a gradient descent algorithm to determine the optimal patient position. We investigated the performance of the DGPP algorithm for reaching adequate HNC target coverage for the patented positioning system for radiotherapy.^27^

## 2 METHODS

### 2.1 Patient data set

We used the data of 45 HNC patients consented for research and treated with IMPT at our institute between December 2021 and October 2023, to examine the performance of the DGPP algorithm discussed in this work. The included patients received definitive proton therapy with a primary clinical target volume (CTV) of 70 Gy(RBE) and an elective CTV with a prescribed dose of 54.25 Gy(RBE), RBE = 1.1, delivered over 35 fractions. The clinically approved IMPT treatment plans were robustly optimized in RayStation (Raysearch ltd, Stockholm, Sweden) with 3 mm setup error and 3% range error on a dose grid with voxel size of 3 × 3 × 3 mm^3^, following our institute's protocol.^28^, ^29^ Per patient five to seven weekly repeat diagnostics CTs (rCTs) (Siemens SOMATOM Confidence, Siemens Healthineers, Germany) obtained according to our institute's protocol for quality assurance during the entire course of the treatment were available. As part of the treatment quality assurance process these weekly rCTs were rigidly registered to the pCT using 6 DOFs (translations and rotations) based on anatomical features in the treatment planning system (TPS) RayStation. Nine patients underwent an IMPT plan adaptation during the course of their treatment.

### 2.2 Dose computation algorithm

For fast dose computation, we used Moqui, an open‐source GPU‐accelerated memory‐efficient MC code for computing proton dose,^26^ and a Linux server with 512 GB RAM and 2 Nvidia A40 (48 GB) GPUs. A beam model specific to our institute's treatment machine (IBA Proteus® PLUS, IBA, Belgium) was developed using available measurement data and validated with the plan quality assurance measurements of HNC patients treated with proton therapy to our institutional protocol.^28^, ^30^ For details we refer the reader to the Supplementary materials. For the conversion of CT Hounsfield units to materials in Moqui, the material properties of 24 Schneider materials collected from the publicly available database of MCsquare were used.^31^, ^32^

We compared Moqui dose computations to the clinical TPS RayStation by performing a global 3D gamma analysis^33^ with a 2%/2 mm dose/distance threshold for the primary treatment plan doses for the group of 45 HNC patients described above. The gamma analysis was performed using the python package PyMedPhys.^34^ The treatment plan doses computed in RayStation were used as reference. The dose cutoff below which gamma is not evaluated was set to 10% of the global maximum and the search distance was not constrained. The doses in Moqui were computed with 1000 and 10,000 particles per history (pph). Lower pph values correspond to larger numbers of simulated histories resulting in higher statistical precision, but with increased computation time.

### 2.3 DGPP algorithm

Our algorithm for DGPP uses Nesterov accelerated gradient descent^35^ to find the optimal position based on dose. We defined the optimal position as the minimizer of the objective function

(1) $$\begin{array}{cc} f & =-\left(0.7*V95{\%}_{\mathrm{CTV}\;70\;\mathrm{Gy}(\mathrm{RBE})+1\;\mathrm{mm}}\right.\\ & \;\left.+0.3*V95{\%}_{\mathrm{CTV}\;54.25\;\mathrm{Gy}(\mathrm{RBE})+1\;\mathrm{mm}}\right), \end{array}$$

with V95% the relative volume receiving at least 95% of the prescribed dose, such that minimizing f corresponds to maximizing target coverage. A larger relative weight was assigned to the primary CTV 70 Gy(RBE) to reflect the higher clinical priority of adequate coverage of the CTV 70 Gy(RBE) compared to the elective CTV 54.25 Gy(RBE). A uniform 1 mm margin was applied to achieve robustness against residual positioning uncertainties.^29^ A smaller margin than the 3 mm setup uncertainty used during treatment plan optimization was chosen because the DGPP algorithm explicitly recomputes dose on the anatomy of the day for different candidate isocenter positions. Consequently, dosimetric effects of interfractional anatomical changes and treatment isocenter shifts are directly incorporated into the optimization, reducing the need for larger setup margins. Furthermore, the 1 mm margin serves as an optimization structure that facilitates full target coverage by guiding the optimization towards positions that provide robust dose coverage at the CTV boundary. To find the minimizer of f, in each iteration t the patient position x=(x,y,z) was updated by

(2) $$x_{t}=x_{t-1}-v_{t},$$

where

(3)

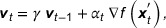

with γ=0.3 the momentum parameter,

(4) $$\alpha_{t}=\alpha_{0}\;\left(1.1-0.1t\right),\alpha_{0}=2\cdot 10^{2}{\mathrm{mm}}^{2}$$

the step size parameter with t≤8, and $\nabla f(x_{t}^{\prime })$ the gradient of f at the partially updated position

(5) $$x_{t}^{\prime }=x_{t-1}\;-\gamma \;v_{t-1}.$$

The components of the gradient of f at position $x^{\prime }\;=\left(x^{\prime },y^{\prime },z^{\prime }\right)$ were determined by

(6) $$\nabla_{i}f\left(x^{\prime }\right)=\frac{f\left(x^{\prime }+\varepsilon \hat{ı}\right)-f\left(x^{\prime }-\varepsilon \hat{ı}\right)}{2\varepsilon }\;,$$

with $i=x^{\prime },y^{\prime },z^{\prime }$, $\hat{ı}$ the unit vector in the direction of the i‐component, and ε=1 mm. The factor (1.1−0.1t) was included so that the gradient descent more easily converges. The gradient descent was terminated when

(7) $$\left|f_{t}-f_{t-1}\right|<0.2\%,$$

with $f_{t}$ the updated value for f in iteration t, or after eight iterations. We observed this condition works reasonably well for reaching convergence. The position among the iterations with the lowest value for f was chosen as the final position.

This process involves multiple dose computations, since for each iteration of the gradient descent the dose needs to be computed seven times: six times to determine the gradient of f in three dimensional space and one time to determine the value for f at the updated position. Therefore, to maintain clinically acceptable runtimes for an online clinical setting, the number of particles per history was set to 75,000. For determining the V95% values during the dose‐guided optimization we wrote a python script that uses the scikit‐rt python package^36^ to convert contours in the structures files exported from the TPS to masks on the CT grid.

### 2.4 Examination of the performance of the DGPP algorithm

**FIGURE 1:**
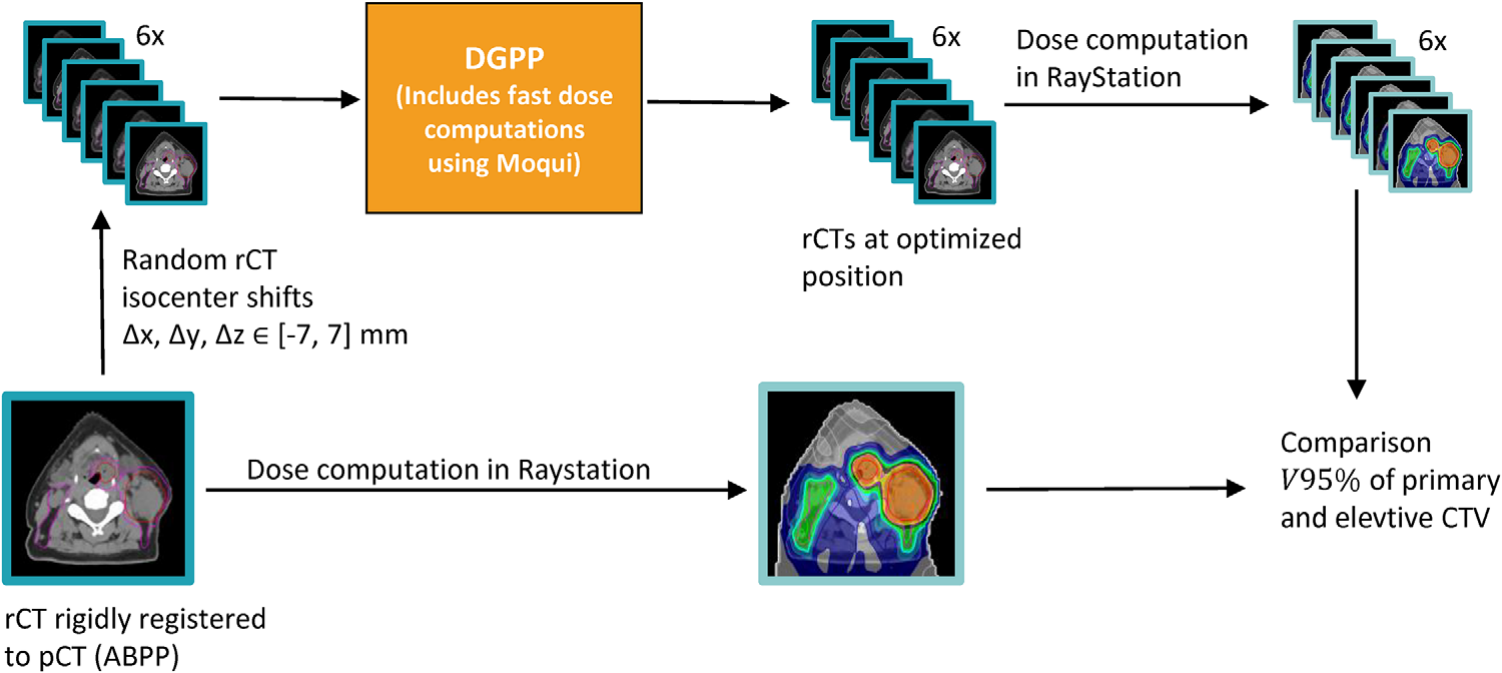
Schematic overview of the workflow followed to examine the performance of the DGPP algorithm.

We examined the performance of the DGPP algorithm by using weekly rCTs from the patient data set described above as surrogates for the daily in‐room images. The positions of the rCTs relative to the plan isocenter were optimized based on dose using the DGPP algorithm. If a patient underwent a plan adaptation, rCTs acquired after the plan adaptation were evaluated using the corresponding adapted plan. The rCT position obtained by 6‐DOFs rigid registration to the pCT for treatment quality assurance we regarded as the patient treatment position following from anatomy‐based patient positioning (ABPP). The workflow applied for each rCT is illustrated in Figure 1. The DGPP algorithm was applied using six initial positions per rCT. These initial positions were obtained by performing random rCT isocenter shifts from the ABPP position. In a clinical workflow in which patients are immobilized using a thermoplastic mask and initial alignments are performed using lasers and two‐dimensional kilovoltage radiographic images, translational corrections based on 3D imaging are expected to be small, generally within 5 mm.^37^, ^38^, ^39^, ^40^, ^41^ Therefore, random rCT isocenter shifts were constrained to ±7 mm in each of the three spatial directions (craniocaudal, lateral, anteroposterior). With this constraint the maximum possible distance in three‐dimension space of an initial position to the ABPP position was 12 mm, corresponding to ±7 mm shifts in all three spatial directions. Doses for the final positions determined by the DGPP algorithm were recomputed in the clinical TPS RayStation. The resulting V95% values of the CTVs were compared to those obtained using ABPP to assess the performance of the DGPP algorithm for reaching good target coverage.

## 3 RESULTS

The primary treatment plan doses computed on the pCT in Moqui compared well to the doses computed in the clinical TPS RayStation. The results for the 2%/2 mm gamma pass rates of the Moqui doses, with the RayStation doses as reference, and the Moqui dose computation times are given in Table. 1. Moqui doses were computed with 1000 and 10,000 pph.

**TABLE 1:** Gamma pass rates and dose computation times of the 45 HNC primary treatment plan doses computed with 1000 and 10,000 particles per history in Moqui.

Particles per history	2%/2 mm gamma pass rate (%)	Computation time (s)
1000	[99.1, 99.8]	[172, 449]
10,000	[97.3, 98.5]	[18, 48]

The corresponding doses computed in the clinical TPS RayStation were used as reference doses in the gamma evaluation.

**FIGURE 2:**
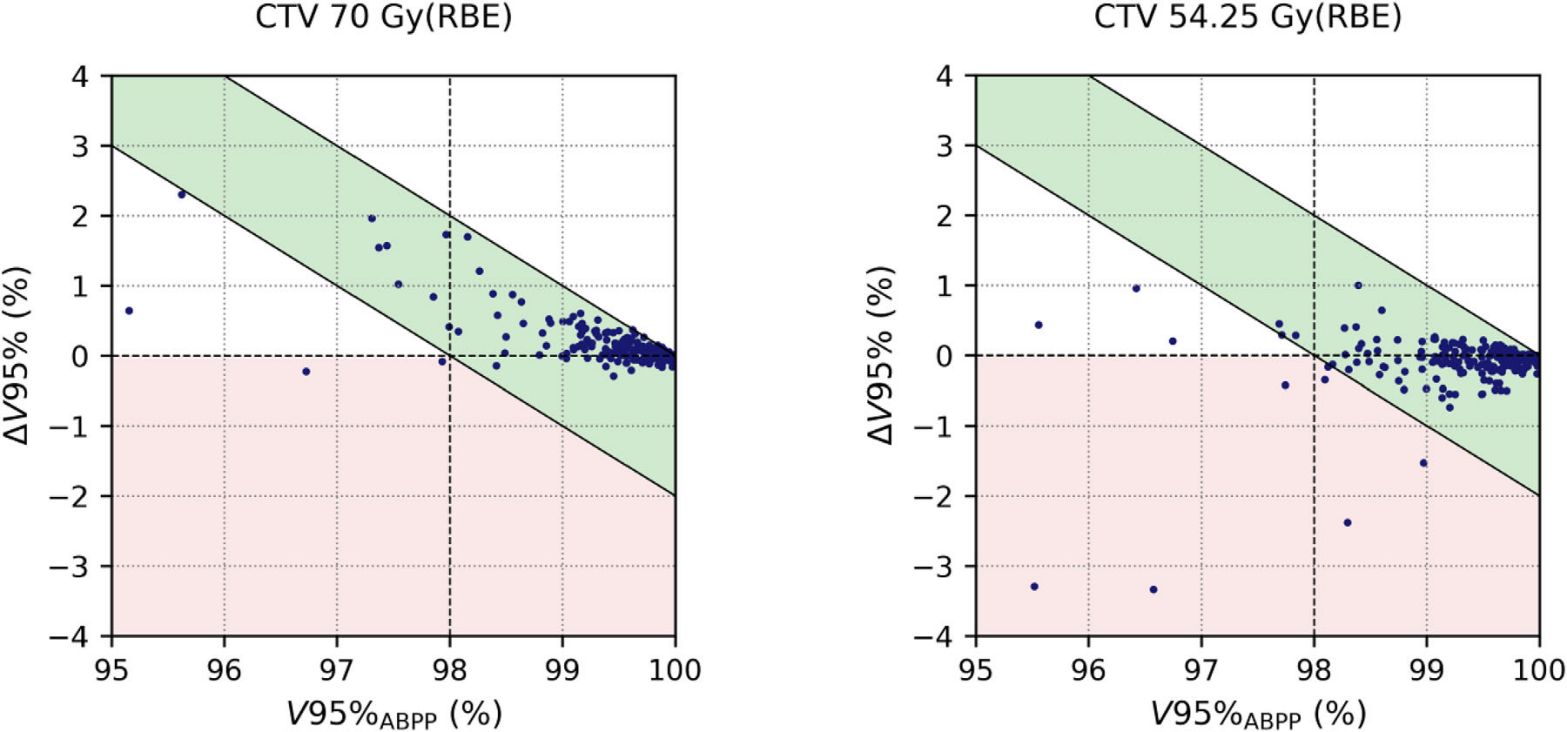
The change in V95% (ΔV95%) by emploing DGPP compared to ABPP for the CTV 70 Gy(RBE) (left) and the CTV 54.25 Gy(RBE) (right). The dots indicate the mean result from the six initials positions per rCT. The green area enclosed by the solid line indicates the region 98%<V95%<100% with DGPP. For dots in the red area the V95% was lower with DGPP compared to ABPP and the clinical goal V95%≥98% could not be met.

**FIGURE 3:**
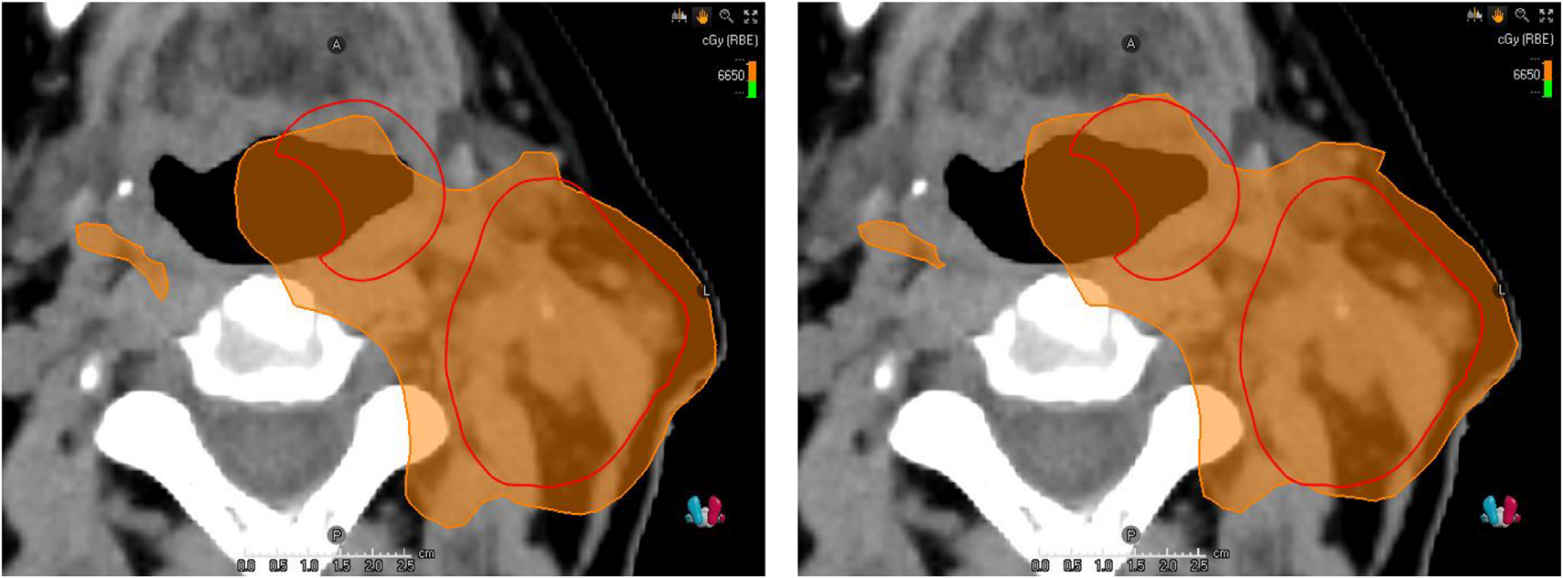
Coverage of the CTV 70 Gy(RBE) (red contour) by the 95% dose level (orange area) of the prescribed dose with ABPP (left) and DGPP (right).

The DGPP algorithm was applied for six initial positions per rCT. We found good convergence. The mean distance of the six initial positions to their mean position ranged from 3 to 8 mm. After applying the DGPP algorithm the mean distance to the mean position was within 2 mm for all rCTs. The distance of the final position following from DGPP to the ABPP position ranged from 0 to 5 mm with an average of (2 ± 1) mm. In Figure 2 the resulting difference in V95% (ΔV95%) between the position obtained with DGPP and the ABPP position is shown for the CTV 70 Gy(RBE) (left) and CTV 54.25 Gy(RBE) (right). Per rCT the mean of the results of the six initial positions is plotted. The standard deviation of ΔV95% per rCT was on average 0.1% for both CTVs and at most 0.6% and 1.8% for the CTV 70 Gy(RBE) and CTV 54.25 Gy(RBE), respectively. For the entire patient cohort, the mean ΔV95% of the CTV 70 Gy(RBE) and 54.25 Gy(RBE) were (0.1 ± 0.3)% and (−0.1 ± 0.4)%, respectively. Eleven cases did not reach our institutional clinical goal V95%≥98% CTV 70 Gy(RBE) with ABPP. Considering only these cases, the V95% of the CTV 70 Gy(RBE) improved on average with (1.0 ± 0.8)%. This resulted in that with dose‐guided optimization seven out of these eleven cases could have reached the V95%≥98% CTV 70 Gy(RBE) clinical goal, as indicated by the green area in Figure 2a. For the rCT for which V95% CTV 70 Gy(RBE) was 95.6% with ABPP, the CTV 70 Gy(RBE) target coverage that could be obtained with DGPP compared ABPP on a representative CT slice is illustrated in Figure 3. A clear improvement can be observed. For two out of the eleven cases, the V95% CTV 70 Gy(RBE) slightly decreased with respect to ABPP, indicated by the red area in Figure 2a, with at most 0.2%, which can be explained by small differences in dose computation between RayStation's dose engine and the Moqui dose engine used by the DGPP algorithm. For four rCTs the V95% of the CTV 54.25 Gy(RBE) decreased considerably, by more than 1%, with dose‐guided optimization and the clinical goal V95%≥98% CTV 54.25 Gy(RBE) could not be met, indicated by the red area in Figure 2b. The standard deviation of ΔV95% CTV 54.25 Gy(RBE) was large for these rCTs, ranging from 0.5% to 1.8%. For all other rCTs the standard deviation of ΔV95% CTV 54.25 Gy(RBE) was within 0.5%. The four rCTs belong to the same patient. The decrease in the V95% of the CTV 54.25 Gy(RBE) was accompanied by an increase in the V95% of the CTV 70 Gy(RBE).

**FIGURE 4:**
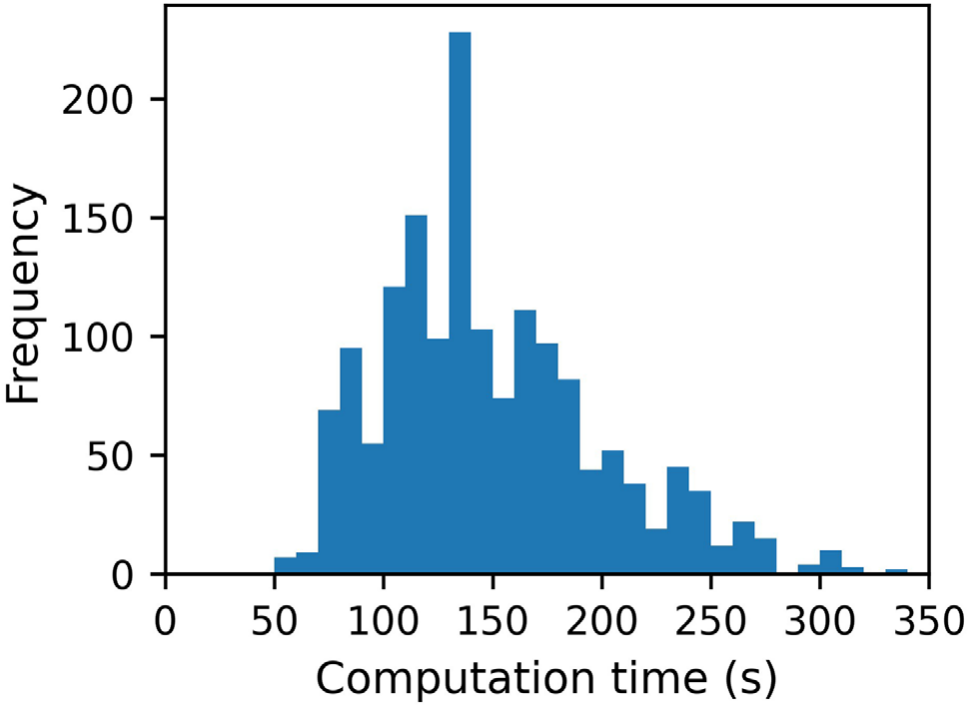
Histogram showing the frequency of the computation times of the DGPP algorithm in bins of 10 s for our data set with 45 HNC patients, five to seven rCTs per patient, and six randomly chosen initial positions per rCT for which we examined the performance of the DGPP algorithm.

On average similar V95% values were obtained with DGPP as with ABPP. A clear improvement, approximately 1% on average, in the V95% CTV 70 Gy was observed for the subset that did not meet the V95%≥98% CTV 70 Gy(RBE) clinical goal with ABPP. Four iterations were on average performed before termination of the gradient descent, either because of convergence as defined by Equation (7) or because the maximum number of eight iterations was reached. The computation time of the DGPP algorithm per initial position is shown in Figure 4, ranging from 53 to 337 s with an average value of (149 ± 51) s.

## 4 DISCUSSION

We examined the performance of a gradient descent algorithm for DGPP using the MC dose engine Moqui. The excellent agreement between Moqui and both measurement data and RayStation supports the suitability of the dose engine for dose‐guided positioning. Although validation was limited to HNC treatment plans, gamma pass rates exceeded the clinical acceptance threshold for all evaluated cases.^30^ The DGPP algorithm showed good convergence in position when starting from different initial positions and performed well in terms of target coverage. The V95% of the primary and elective CTV obtained by DGPP did not differ on average from that obtained with ABPP. However, for the subset of rCTs that did not meet the clinical goal of V95% ≥ 95% for the CTV 70 Gy(RBE) with ABPP, DGPP improved the V95% by approximately 1% on average, allowing most of these cases to reach the clinical target coverage goal. The optimal positions could be determined in less than three minutes on average, suggesting potential feasibility for clinical implementation. These findings demonstrate that automated DGPP can achieve target coverage comparable to ABPP at a clinically relevant timescale in HNC patients treated with IMPT, while improving primary target coverage in cases where the clinical target coverage goal is not achieved with ABPP. Improved target coverage may reduce the dosimetric impact of interfractional anatomical changes and could complement adaptive proton therapy workflows.^42^

Previous studies by Cheung et al.,^13^ Haehnle et al.,^14^ and Kurz et al.^15^ demonstrated that optimization of patient position can improve dosimetric outcomes without replanning. While the overall objective is similar, the implementation differs substantially between studies. In particular, Kurz et al.^15^ employed a predefined set of sampled positions combined with dose interpolation, resulting in a fixed number of dose evaluations, to perform multicriteria optimization including dosimetric objectives for OARs, whereas the present work used iterative optimization with direct MC dose recomputation and focused on target coverage. Therefore, direct comparisons of computational performance between studies should be interpreted with caution.

The present work differs from previous studies through its use of fast MC dose recomputation and iterative optimization of patient position. Compared with interpolation‐based approaches,^14^, ^15^ it requires a larger number of dose computations during optimization. The primary objective of this study was not to minimize the number of dose evaluations, but rather to investigate whether direct MC dose computations could be incorporated into an online positioning workflow while maintaining high dosimetric accuracy. Our method performs direct MC dose recomputation at every candidate position evaluated during the optimization process. This choice was made to ensure that positioning decisions were based on the computed dose distribution itself rather than on interpolated dose estimates, thereby avoiding an additional approximation step in the workflow. While this increases the computational burden, it provides a consistently high‐accuracy dose evaluation throughout the optimization process. The optimization strategy should therefore be viewed as a trade‐off between computational efficiency and dosimetric accuracy. Although more dose computations were required than in interpolation‐based approaches, optimization was completed within approximately three minutes on the available hardware. Consequently, the computational cost did not preclude potential integration into an online positioning workflow. Future work could investigate whether interpolation of MC‐generated dose distributions can further reduce computation times while maintaining the positioning accuracy observed in this study.

Although the observed computation times suggest that DGPP could potentially be incorporated into an online workflow, several practical aspects remain to be investigated before clinical implementation. In the present study, only the optimization and dose computation times were evaluated. In a clinical setting, additional steps such as image review, assessment of the proposed correction, and routine treatment‐room procedures would also contribute to the overall treatment time. Consequently, the impact of dose‐guided positioning on workflow efficiency may differ from the computation times reported here. Furthermore, implementation in routine clinical practice would require integration within a validated and clinically approved software environment. Future prospective studies are needed to assess workflow integration, user acceptance, and the overall clinical benefit of online dose‐guided positioning.

Developments in computing power and GPU‐based algorithms enabled the use of a fast MC dose engine for dose recomputation by the DGPP algorithm presented in this work. However, to maintain clinically acceptable computation times, a trade‐off was made between statistical precision and computation duration. In future work, we aim to further accelerate Moqui so that the optimization time of the DGPP algorithm can be reduced while improving the statistical precision of the dose computations during gradient descent. This could be achieved, for example, by further optimizing Moqui for the GPU server architecture used in this study, reusing particle transport information, or incorporating AI‐based methods to convert low‐statistics dose computations into high‐statistics dose distributions.^20^ In addition, further fine‐tuning of the gradient descent parameters and convergence criteria may reduce the number of required iterations and therefore further decrease computation times.

For four rCTs, all from a single patient, DGPP resulted in a reduction in V95% of the elective CTV compared with ABPP, reflecting a trade‐off favoring coverage of the primary CTV. The choice of weights in the objective function of Equation (1) prioritized improving V95% of the primary CTV, for which adequate dose coverage is of greater clinical importance. In this regard, several studies suggest that the prescribed dose to the elective CTV may be safely lowered.^43^, ^44^, ^45^, ^46^ Future refinements of the objective function may enable a more balanced optimization of the primary and elective target volumes. For example, clinical goal‐based weighting factors could be introduced to modulate the contribution of each term once the corresponding clinical goal has been achieved. In addition, a major advantage of DGPP is that it automatically provides insight into the expected treatment dose distribution before delivery, whereas with ABPP the dose distribution on the anatomy of the day remains unknown. Trade‐offs between target coverage of the primary and elective CTVs, or between target coverage and OAR dose, would therefore become visible before treatment delivery and could be incorporated into treatment decision‐making and quality assurance procedures. Furthermore, the impact on cumulative delivered dose could be assessed through dose accumulation during the treatment course.^47^, ^48^, ^49^, ^50^

A limitation of the present work is that it is a retrospective study using weekly rCTs rather than daily in‐room images acquired for patient positioning. In the current clinical workflow, rCTs rigidly registered to the planning CT are used as a representation of the patient's weekly anatomy to assess the dosimetric impact of anatomical changes on the treatment plan.^29^, ^51^ We therefore considered them suitable for an initial evaluation of DGPP performance. A further limitation is that the optimization objective focused exclusively on target coverage. Kurz et al.^15^ demonstrated that dose‐guided position optimization including dosimetric objectives for OARs can result in enhanced OAR sparing in HNC patients. In future work, the objective function optimized by gradient descent could be expanded to include OAR dose metrics. It may also be interesting to include cumulative dose objectives, thereby shifting the focus toward identifying the position that best supports the overall treatment course. Additionally, rotational DOFs were not included in the optimization. Although these could be incorporated similarly to translational DOFs, doing so would increase the number of required dose computations and therefore the overall computation time. The potential dosimetric benefit of including rotational DOFs was not evaluated in the present study.

Another challenge for future clinical implementation is the availability of accurate target contours on the image used for dose‐guided positioning. Existing studies have demonstrated the importance of contour accuracy in adaptive proton therapy.^52^, ^53^, ^54^, ^55^, ^56^ Although contour propagation methods can provide contours of acceptable quality for many structures, manual corrections are often recommended. Smolders et al.^55^ demonstrated that the dosimetric impact of using automatically propagated target contours in plan reoptimization instead of manually delineated contours can be substantial. Even though a 1 mm margin was added to the target structures to ensure target coverage robustness during optimization, further work is still needed to determine how contour uncertainties affect dose‐guided positioning performance in the workflow presented here.

## 5 CONCLUSION

This study demonstrated that an automated DGPP algorithm based on gradient descent and the MC dose engine Moqui can optimize patient position for target coverage at near‐clinically acceptable timescales. Similar or improved target coverage compared with ABPP was achieved for HNC patients within an average computation time of approximately three minutes, suggesting that DGPP has potential for clinical implementation in pre‐treatment patient positioning workflows. Further work is warranted to enable routine clinical implementation.

## CONFLICT OF INTEREST STATEMENT

The authors declare no conflicts of interest.

## Supporting information




Supporting Information
