# Supplementary material for: Towards automated dose‐guided patient positioning at clinical timescale in head‐and‐neck cancer proton radiotherapy
Source: Med Phys. 2026 Jul 28;53(8):e70591. doi: 10.1002/mp.70591 (PMC13411819; doi:10.1002/mp.70591)
Supplement: Supplementary file 1 — Supporting Information [file MP-53-0-s001.pdf]

Details on the Moqui beam model development for our institute's treatment machine

Fitting of beam model parameters

A beam model for Moqui was developed for our institute's treatment machine (IBA Proteus® PLUS, IBA, Belgium). The beam model parameters include the beam energy, energy spread, spot size parameters, and the number of particles per monitor unit (MU). These parameters were determined for nominal energies ranging from 70 MeV to 225 MeV, corresponding to the clinically available energy range of our treatment machine, with steps of 10 MeV by fitting to available measurement data. We used integral depth doses (IDDs) measured in water using StingRay (IBA Dosimetry, Germany), in-air spot profiles measured with Lynx PT (IBA Dosimetry, Germany), and in-water absolute dose measurements obtained using a PPC05 Plane Parallel Chamber (IBA Dosimetry, Germany). The in-air spot profiles were measured at different positions along the beam axis, namely at -189, -100, 0, 100 and 230 mm relative to the isocenter. For the absolute dose measurement a field of 10x10 cm^2 and spot spacing of 2.5 mm was created per energy layer and the dose was measured at the field center at a depth corresponding to halfway between 1 cm and the midpoint of the Bragg-peak maximum. For each nominal energy, the beam energy and energy spread were adjusted until the depth of the 80% and 20% distal dose fall-off (R80 and R20, respectively) of in-water computed IDD curves corresponded to the measured ones. For all measured energies, the difference in R80 and R20 between the computed and measured IDDs were within 0.1 mm. The spot size parameters were obtained by matching the standard deviation of single Gaussians fitted to the measured and computed in-air spot profiles in the two directions perpendicular to the beam at five positions along the beam axis covering a distance of 419 mm. The difference in standard deviation per direction and position was at most 0.2 mm. The number of particles per MU was rescaled for each nominal energy such that the computed dose corresponded to the absolute dose measurement. The difference between the computed and measured absolute point dose at the center of the 10x10 cm^2 field in water was within 0.04% of the measured value.

Beam model validation with plan quality assurance measurements

We validated the beam model with the plan quality assurance (plan QA) measurements of 10 HNC patients treated with proton therapy according to our institutional protocol. Each treatment plan consisted of four to five beam orientations. The dose was measured per beam at three different depths in water-equivalent material using RW3 Slab Phantom (PTW Freiburg GmbH, Germany) and MatriXX PT (IBA Dosimetry, Germany). We performed a 2D gamma analysis with a 2%/2 mm dose/distance threshold criterion in myQA v2022-001 (IBA Dosimetry, Germany) comparing the doses computed in Moqui to the measured doses. The measured doses were used as reference. A dose threshold of 10% and a maximum search distance of 7.5 mm was used. The statistical precision of Moqui dose computations is determined by the particles per history (pph) parameter, which we set to 1000. The resulting 2%/2 mm gamma pass rates, averaged over beams and depths are shown in Tab. 1. For nine out of the ten patients, the average gamma pass rate comparing the Moqui doses with the measured doses was above 99.5%, while for one patient it was 98.2%. The results were comparable to those obtained from the gamma analysis comparing the doses computed in the clinical treatment planning system RayStation (Raysearch ltd, Stockholm, Sweden) with the corresponding measured doses (Tab. S1).

Patient	Average 2%/2mm gamma pass rate (%)	
	Moqui	RayStation
**1**	99.5	99.6
**2**	100.0	99.9
**3**	99.8	99.9
**4**	98.2	97.7
**5**	99.9	99.8
**6**	100.0	99.9
**7**	99.8	99.2
**8**	99.9	99.8
**9**	100.0	99.9
**10**	100.0	99.9

Table S1: The 2%/2 mm gamma pass rates of Moqui and RayStation plan QA dose computations averaged over the beams and measured depths for 10 HNC treatment plans. The measured doses were used as reference.
